# Alkali-Activated Slag Repair Mortar for Old Reinforced Concrete Structures Based on Ordinary Portland Cement

**DOI:** 10.3390/ma18102272

**Published:** 2025-05-14

**Authors:** Danutė Vaičiukynienė, Agnė Liudvinavičiūtė, Reda Bistrickaitė, Olha Boiko, Vilimantas Vaičiukynas

**Affiliations:** 1 Faculty of Architecture and Civil Engineering, Kaunas University of Technology, Studentų st. 48, LT 51367 Kaunas, Lithuania; agne.liudvinaviciute@gmail.com (A.L.); reda.bistrickaite@ktu.lt (R.B.); olia.bojkoyt@gmail.com (O.B.); 2 Scientific Research Institute for Binders and Materials, Kyiv National University of Construction and Architecture, Povitrianyh Syl ave., 31, 03037 Kyiv, Ukraine; 3 Faculty of Environmental Engineering, Lietuvos Inžinerijos Kolegija/Higher Education Institution, Tvirtovės al. 35, LT 50155 Kaunas, Lithuania; vilimantas.vaiciukynas@hotmail.com

**Keywords:** OPC repair mortar, alkali-activated slag repair mortar, biomass bottom ash aggregate

## Abstract

In this study, alkali-activated mortars were prepared using two different types of fine aggregates: natural sand and biomass bottom ash. These mortars were used as a repair material for structures constructed using old reinforced concrete structures based on Ordinary Portland cement (OPC). Experimental studies have shown that the alkali-activated slag mortar with biomass bottom ash (BBA) from the bubbling fluid bed meets the repair mortar class R1 according to EN 1504-3. The suitability of such repair mortar is determined by the good adhesion properties of the alkali-activated slag binder to old OPC concrete. The adhesion after 28 days was 0.31 MPa and the samples broke off at the repair matrix. The AAC/BBA repair mortar had a compressive strength of 18.69 MPa, the shrinkage due to drying deformations consisted of 0.1903% after 28 days. Alkali-activated slag mortars are effective in repairing, renewing and rebuilding damaged OPC concrete structures.

## 1. Introduction

Repairing reinforced concrete structures is subject to a wide range of chemical, physical, mechanical and biological processes that damage the integrity of the structure and degrade its performance. A widely used method of repairing reinforced concrete structures is the use of OPC-based repair mortars. For example, the OPC cement-based mortar is often used as repair material [1]. However, the high consumption of OPCs on a global scale has an impact on climate change. An alternative to OPCs is the use of alkali-activated binders, which can reduce the CO_2_ footprint. In addition, various industrial by-products based on alumina and silicate compounds (active forms) are reused in alkali-activated systems. Kryvenko et al. [2] explained that alkali-activated binders have high mechanical and durability properties due to their mineral composition. In alkali-activated systems, free Ca(OH)_2_ and ettringite are not formed, and highly alkaline calcium silicates are replaced by low-alkaline calcium silicates and the hydration products of zeolitic phases. Alkali-activated materials have great potential for use as repair materials for OPC concrete, according to Geraldo et al. [3]. Alkali-activated reparation materials were found to be prepared from metakaolin and a silica fume-based precursor with a sodium hydroxide alkaline activator. River sand was used as a fine aggregate. This formulation has a strength of 30 MPa, which resulted in good adhesion between the concrete to be repaired and this alkali-activated mortar. According to Fan et al. [4,5], the adhesive strength of a geopolymer and OPC concrete is the main role of repair materials. This adhesive strength is closely related to the amount of initial materials. The best precursor composition for the production of repair materials has been found to be 50% slag and 50% metakaolin. This composition resulted in the highest volume density values, which are closely related to mechanical properties and durability. Good adhesion is due to the reaction between the alkali-activated mortar and the old, repaired concrete. In this case, certain gels are formed, such as C/N-S/A-H. These hydration products result in a denser contact zone, which has a positive effect on mechanical properties. Yusslee et al. [6] found that alkali-activated binders made from a blended precursor (more than one component) have a higher adhesive strength compared to a precursor made from only one component. This could be explained by the formation of more types of hydration products such as C–S–H, C–A–S–H, N–A–S–H gel and/or zeolitic phases. Gomaa et al. [7] investigated the use of five different types of alkali-activated fly ash to produce a repair material suitable for OPC concrete repair. This repair material was cured at an ambient temperature. Pull-out tests showed an adhesion of 5.8 MPa between the alkali-activated fly ash and the OPC concrete. The adhesion was slightly improved due to the higher calcium content of the system. CASH was formed as a new chemical compound in the contact zone.

Durability properties such as high temperature resistance and resistance to chloride penetration into the adhesive interface were better for the alkali-activated repair materials than for the OPC materials. Zhang et al. [8] produced geopolymer from a combined slag and fly ash precursor. Different doses of slag and fly ash in the composition of a precursor were used. The old concrete based on Ordinary Portland cement was repaired with this geopolymer and high temperature resistance was investigated. The geopolymer coatings that were applied to the old concrete were found to have excellent bonding properties and can be used as a repair material for ordinary concrete repair. These geopolymer repair materials have good adhesive bond strength at high temperatures. The best mechanical and microstructural properties were obtained when the precursor contained 50% slag and 50% fly ash. Similar results were found by Fan et al. [5]. The repair of old OPC concrete using a sustainable slag and fly ash geopolymer led to the conclusion that the most effective repair material was made from a mixture of 50% slag and 50% fly ash. The reactions found at the interface between the OPC concrete and the geopolymer were C-S-H, C-A-S-H and N-A-S-H gels. Durability, such as resistance to high temperature, chloride penetration and shrinkage, was closely related to the composition of the precursor and was best with the samples made of 50% slag and 50% fly ash. Coppola et al. [9] investigated an environmentally friendly alkali-activated slag-based mortar for the repair of existing masonry buildings and old concrete. The activator to the precursor ratios were found to be significant. A higher content of alkali activators in the system resulted in an improvement of the mechanical properties of the mortar. On the other hand, higher shrinkage due to drying was due to a higher alkali content. A higher alkali content led to a higher content of dissolved silica and aluminum, resulting in an increase in the amorphous phase, which is closely related to higher shrinkage. In the study [10], alkali-activated repair mortars were prepared using waste glass powder and slag as a precursor and glass cullet as a fine aggregate. The glass cullet reduced the drying shrinkage compared to the samples prepared using river sand as the fine aggregate, with the best composition being 50% waste glass powder in the precursor and 100% glass mortar as the fine aggregate.

In this study, blast furnace slag was used as a precursor for aluminum and silica with sodium hydroxide solution was used as an activator. The aim of this study is to analyze the performance of an alkali-activated slag mortar for the repair of a reinforced OPC concrete beam. Two types of fine aggregates were used to produce the alkali-activated slag mortar: natural sand and biomass bottom ash (BBA).

This research contributes to the development of an environmentally friendly repair mortar based on by-products such as ground granulated blast furnace slag as a precursor and biomass bottom ash as an aggregate. It also saves natural resources, reuses production waste and ultimately creates a new type of mortar. The utilization of locally sourced waste and by-products has the potential to reduce transportation and energy expenditure; furthermore, the manufacturing technology is less expensive than traditional binders.

## 2. Methods and Materials

### 2.1. Initial Materials

For the reference repair mortar, OPC CEM I 32.5 R was chosen as a binding material. The X-ray fluorescence (XRF) analysis shows a predominance of CaO, SiO_2_ and Al_2_O_3_ in OPC (Table 1). The fineness of OPC used was 392 m^2^/kg.

Blast furnace slag has been used as a calcium and silica precursor with specific surface area of 219 m^2^/kg. In this case, SiO_2_, CaO, Al_2_O_3_ and MgO accounted for the largest proportion of the total oxides. According to the X-ray diffraction (XRD) analysis, calcite, quartz and hydrotalcite were determined as crystallin compounds.

For an aggregate, two types of sand were selected. The first type of aggregate was standard quartz sand, in accordance with LST EN 12620:2003+A1:2008 [11]. The chemical composition showed that SiO_2_ dominates in sand, and it accounts for more than 97% of it (Table 1). The second type of aggregate was biomass bottom ash (BBA) from a bubbling fluidized bed.

**Figure 1:**
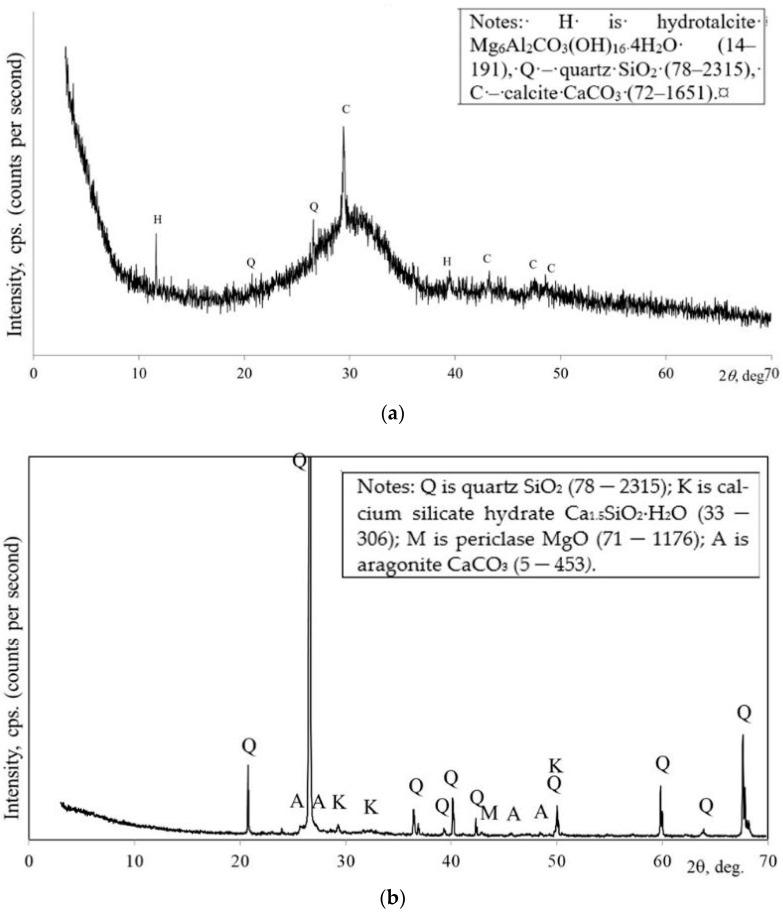
Mineral composition of slag (**a**) and biomass bottom ash (**b**) according to XRD analysis.

According to mineral composition, hydrotalcite, quartz and calcite are detected as crystalline compounds in the slag (Figure 1a). In the case of amorphous SiO_2_, a hill appears in the XRD diagram at 20–35 2q degrees of 2q. Slag is a suitable precursor for the production of alkali-activated binders due to its high content of silicon and aluminum compounds in active form [12]. Quartz, calcium silicate hydrate, periclase MgO and aragonite are the main minerals of biomass bottom ash (Figure 1b). A similar mineral composition of this ash was determined by Luna-Galiano et al. [13].

A NaOH solution (9.7 M), as an activator, was prepared from NaOH pellets and water.

**Figure 2:**
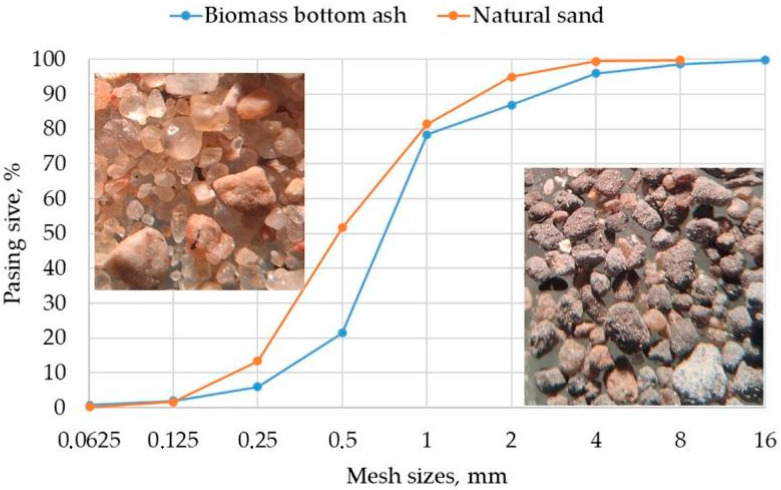
Granulometric distribution of fine aggregates: sand and biomass bottom ash, for repairing mortars.

The natural sand of a 0/4 fraction and with a bulk density of 1560 kg/m^3^ was used as a fine aggregate. The granulometric distribution of particles is shown in Figure 2. Similar particle granulometric distribution was seen in biomass bottom ash. The slightly higher bulk density of biomass bottom ash was 1624 kg/m^3^. This ash is produced from biomass. In a bubbling fluidized bed boiler, silica sand is added at high temperatures to maintain thermal inertia. The burning fuel mixes with this sand to produce a waste-type ash sand [14]. Optical microscopy images (Figure 2) show that after the combustion process a darker color change in the sand can be observed and a small amount of fine particles appear on the sand grains.

Thus, in this study, biomass bottom ash from the fluidized bed combustion of biomass was used as a fine aggregate. A previous study [15] analyzed cement mortar (adhesive) with this type of ash. The results showed that the adhesive mortar with BBA, which replaced the natural sand, increased the adhesive strength and the interfacial contact between the adhesive and the ceramic tile.

### 2.2. Preparation of the Repairing Mortar Mixtures

**Figure 3:**
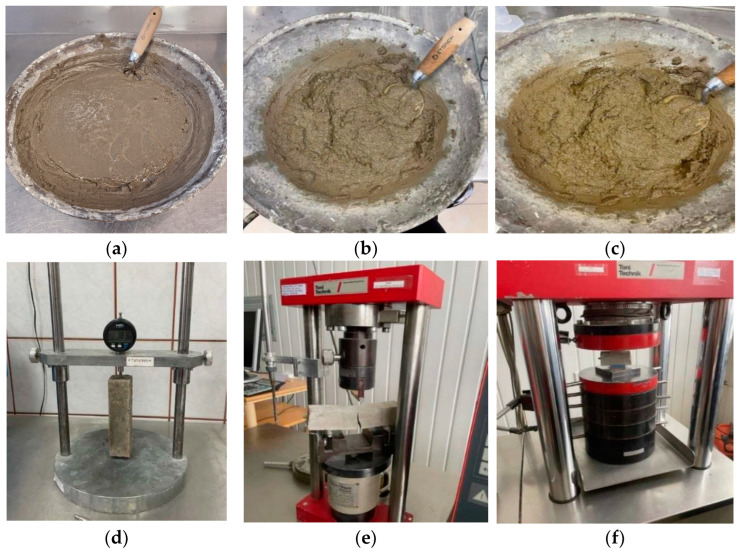
Repair mortar with Portland cement (**a**), alkali-activated slag (**b**) and repair mortar with alkali-activated slag (**c**). Investigation methods of mortars: shrinkage deformations (**d**), flexural strength (**e**) and compressive strength (**f**).

The reference repair mortar (OPC/sand) was prepared as follows: first, the OPC was mixed with the natural sand aggregate and then filled with water (Figure 3).

The second (AAS/Sand) and third (AAS/BBA) mortars’ slag was mixed with sand or bottom ash and filled with a sodium hydroxide solution to produce alkali-activated mortars (Figure 3). An RUBIMIX-9N portable mortar mixer with a speed of 780 rpm was used for the mixing. After careful mixing, the mortars were cast in molds of the size of 4 × 4 × 16 cm and on the old OPC reinforced concrete beam (Figure 3). These samples were used for the determination of mechanical properties and to evaluate shrinkage drying deformations during the hardening process. An old concrete beam was added with repair mortar and after that the beam with repair mortar was covered with polyethylenic material to prevent water evaporation and stored at an ambient temperature for 28 days. This curing duration was chosen in accordance with EN 1015-11 [16].

The amount of initial materials for the investigated mortars is given in Table 2.

### 2.3. Experimental Techniques

Chemical composition was determined according to X-ray fluorescence (XRF) with a Bruker X-ray S8 Tiger WD spectrometer (Karlsruhe, Germany). Mineral composition was evaluated according to X-ray diffraction (XRD) with a D8 Advance diffractometer, Bruker AXS (Karlsruhe Germany). The database used for peak identification was PDF-2. The particle size of the aggregates was determined by sieving the materials through sieves according to LST EN 12620: 2008 [11]. The specific surface area of the OPC and the ground slag was analyzed using a Blaine apparatus according to EN 196-6:2018 [17]. The determinations of shrinkage due to drying and expansion were made on 40 × 40 × 160 mm prismatic samples with raps at the ends, according to EN 12390-16 [18]. Shrinkage was measured on three samples of each type and the mean was calculated and averaged. The flexural strength of the mortars was obtained from three prisms (40 × 40 × 160 mm) and the compressive strength was obtained from six cubes (d = 40 mm; h = 40 mm) as an average value. The strength of the samples was determined using a ToniTechnik 2020 hydraulic press (Berlin, Germany) by loading the samples to failure (EN 12390–4) [19] in a force-controlled mode at a stress rate of 0.8 MPa s^−1^ for the compression tests and in a displacement-controlled mode at 0.5 mm s^−1^ for the flexural tests. The determination of the volume density of mortar samples is given in LST EN 12390-7:2009 [20]. The adhesion of the materials was tested using a PROCEQ DYNA Type Z16E pull-out tester according to EN 1542-1999 [21] and the arithmetic mean of the three samples was calculated. The microstructures of the natural sand and biomass bottom ash were assessed using optical microscopy images taken with an optical microscope from CETI (Brussels, Belgium). The microstructures of the biomass bottom ash and mortars were examined by scanning electron microscopy using FEI Quanta 200 FEG equipment (Hillsboro, OR, USA).

## 3. Results and Discussion

**Figure 4:**
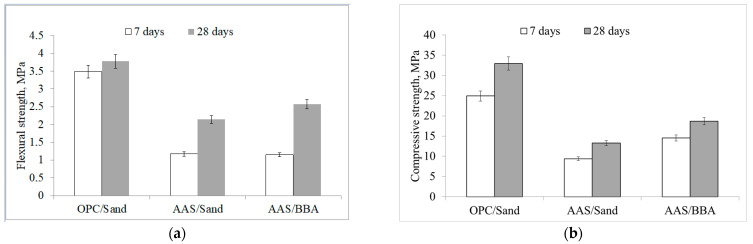
Flexural (**a**) and compression strengths (**b**) of repairing mortars after 7 and 28 days.

The mechanical properties of the mortars tested were first determined. An analysis of the flexural and compressive strength results shows that OPC/sand mortars have the highest values, while alkali-activated mortars have slightly lower values after 7 and 28 days of hydration (Figure 4). After 28 days, the AAS/BBA mortar reached a flexural strength of 2.57 MPa, whereas another type of alkali-activated slag mortar, such as AAS/sand, had a slightly lower flexural strength of 2.14 MPa (Figure 4a).

Similar flexural strengths of 2.5–3.4 MPa were found by Atiş et al. [22] for alkali-activated slag mortar after 28 days. The same trend is true for compressive strength. The highest compressive strengths (32.97 MPa) were for OPC/sand mortar, followed by AAC/BBA (18.69 MPa) and finally AAC/sand mortar (13.25 MPa) (Figure 4b). The strength results are proportional to the volume density of the mortar samples. The OPC/sand mortar had the highest density of 2098 kg/m^3^ and the alkali-activated slag mortars had the lowest densities of 1936 kg/m^3^ and 1894 kg/m^3^ for the AAC/BBA and AAC/sand mortars, respectively (Table 3).

**Figure 5:**
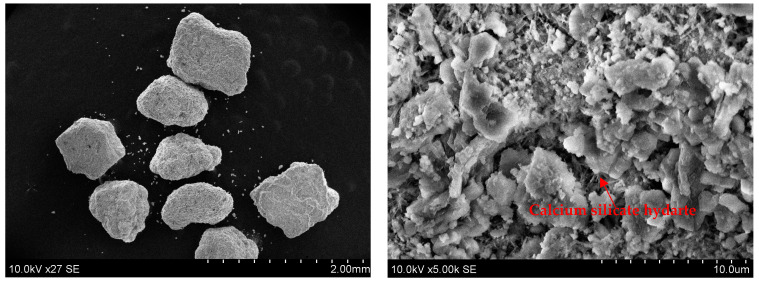
SEM images of biomass bottom ash with different enlargements.

The higher mechanical properties of the AAC/BBA mortar compared to the AAC/sand mortar can be explained by the microstructure and mineral composition of the BBA. Scanning electron microscopy (SEM) was used to determine the microstructure of the BBA (Figure 5). During the combustion process, fine particles coat the sand particles and form new chemical compounds, resulting in a coarse sand particle surface. A similar counting layer on the sand particles after combustion was detected by Schlupp et al. [23]. This rough particle surface results in good adhesion to the binder matrix, e.g., the alkali-activated slag. Another reason for the higher compressive strength may be the formation of a calcium silicate hydrate (CSH) on the surface of the sand particles. The presence of CSH was determined according to XRD analysis (Figure 1b) and by using SEM (Figure 4). The CSH particles are fibrous and were detected on the surface of the sand particles after combustion [24]. The newly formed CSH can act as crystallization centers for the alkali-activated slag and accelerate the geopolymerization of the alkali-activated slag matrix, resulting in an increase in the mechanical strength of these binders. A similar effect of CSH seeding has also been found in previous studies by Puligilla et al. [25]. C-S-H seeds have been found to act as nucleation sites for C-S-H/C-A-S-H. The chemical composition of BBA is significant as well. Table 1 shows that the BBA contains CaO, Na_2_O and K_2_O in its chemical composition. The sum of these oxides amounts to 27.94% and increases the alkaline environment of the binder. This may have a positive effect on the reactivity of BBA, as more geopolymerization products and more compact structures are formed. Thus, K_2_O and CaO can have a positive effect on strength when the blends contain less Na_2_O, as stated by Leong et al. [26] and Vaičiukynienė et al. [27]. The presence of CaO, Na_2_O and K_2_O in the precursor leads to alkali activation with a lower alkali content, according to Peng et al. [28]. 

Another study [15,29] found a similar trend when using BBA as filler. The replacement of BBA with natural sand resulted in an increase in mechanical properties due to the additional reactive component compared to natural sand.

For repairing mortars, dimensional stability is important. The drying shrinkage deformations of the OPC/sand repairing mortar were 0.3210%, while the shrinkage deformations of the alkali-activated mortars were lower, at 0.1903% and 0.3121% for the AAC/BBA and the AAC/sand mortars, respectively, after 28 days of hydration (Table 3). Previous studies [14] have indicated that the drying shrinkage deformations were in the range 0.162–0.2140% for alkali-activated slag.

**Figure 6:**
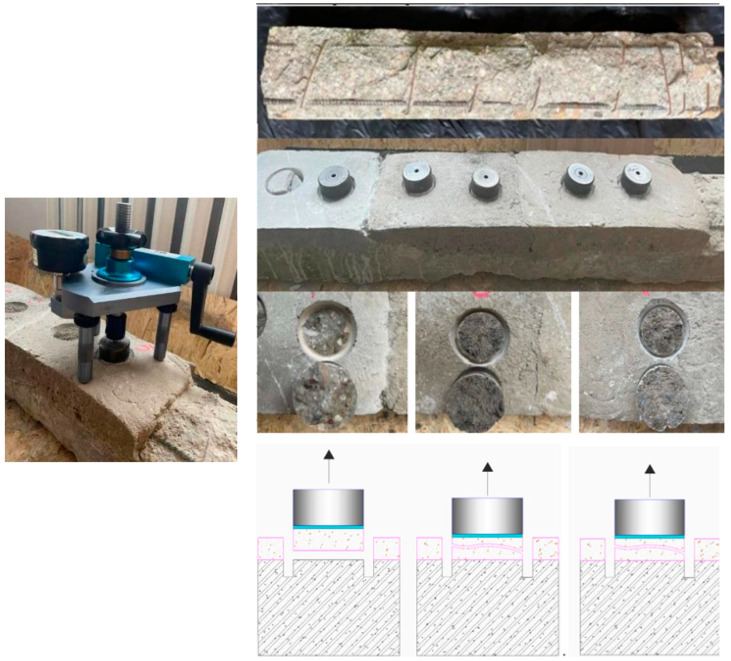
The old OPC concrete beam and visual identification of the type of failure according to a pull-off test (EN 1542) [21].

For the adhesion test, a reinforced concrete beam with a damaged concrete layer was used (Figure 6). The beam was exploited in outdoor conditions and exposed to the natural effects of various chemical and physical processes. Before the repair, organic matter was removed from the surface of the concrete beam. No adhesion-enhancing soil was used to repair the beam. The adhesion between the concrete surface to be repaired and the repair mortars was tested after 28 days. The control sample broke off at a force of 0.59 MPa at the area of the surface to be repaired as shown in Figure 6. This test indicates an insufficient adhesion of the material to the surface to be repaired. The samples of the AAC/BBA and the AAC/sand mortars broke off at 0.31 and 0.29 MPa, respectively. The rupture occurred in a layer of the repair matrix shown in Figure 5. This indicates excellent adhesion of the material to the surface to be repaired, but poor mechanical resistance.

**Figure 7:**
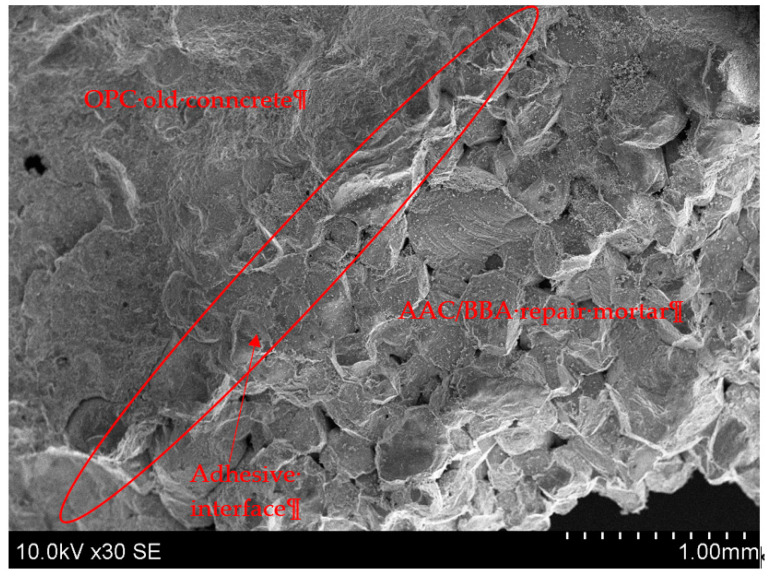
SEM images of adhesive interface with the old OPC concrete and AAC/BBA repair mortar.

According to the experimental results, the AAC mortar has good adhesion to the surface of the old OPC concrete and the contact area is hardly visible (Figure 7). The reason for this good adhesion could be the reaction of the AAC with the old OPC concrete: the Ca(OH)_2_ from the OPC concrete reacted with the silicon and aluminum from the slag to form CASH and/or CNASH, which improved the bond strength. It is likely that alkali-activated binders adhere well to the OPC surface due to the presence of calcium hydroxide Ca(OH)_2_ on the OPC concrete surface, which reacts with the alkali binders. By forming the structure of the geopolymer, Ca^2+^ cations balance the negative charge of Al^3+^ cations in the geopolymer structure. Si^4+^ cations dissolve from the surface of the aggregates in an alkaline environment and participate in the geopolymerization reaction as well. This leads to chemical adhesion between the two surfaces [7].

Thus, this study compared two types of repair mortars based on an alkali-activated slag binder. The first mortar used natural sand as an aggregate and the second used biomass ash. The proposed benefits of this study are the environmental aspect, as two types of waste, slag and BBA, are used, and the economic impact, as these wastes do not need to be landfilled. The AAC/BBA repair mortar could be assigned as a Class R1, according to EN 1504-3 [30].

## 4. Conclusions

The properties of two alkali-activated slag repair mortars made with different aggregates, such as natural sand and biomass bottom ash, were investigated and the results were compared with an OPC mortar. After 28 days, the flexural and compressive strengths were in the range of 2.14–2.57 MPa and 13.25–18.69 MPa, respectively. The additional reactive component of the ashes resulted in higher mechanical properties compared to the mortar made from natural sand. The SEM analysis identified a counting layer on the sand particles after combustion and the XRD analysis confirmed the presence of calcium silicate hydrate. The shrinkage deformations were 0.1903% for the AAC/BBA mortar and 0.3172% for the AAC/sand mortar. The pull-off test showed a strength of 0.31 and 0.29 MPa for the AAC/BBA and for the AAC/sand mortars, respectively. These pull-out test values indicate that the repair mortars have poor mechanical resistance due to the rupture occurring in the repair matrix layer and excellent adhesion to the old OPC concrete due to the reaction of the AAC with the old OPC concrete. Thus, based on the results obtained, this alternative concrete structural repair mortar can be classified as a Class R1 repair mortar suitable for non-structural repair (EN 1504-3).

## Figures and Tables

**Table 1:** Chemical composition of initial materials for XRF analysis (%).

	Oxides										
	SiO_2_	CaO	SO_3_	Al_2_O_3_	Fe_2_O_3_	MgO	K_2_O	Na_2_O	TiO_2_	P_2_O_5_	Other
OPC	19.51	61.32	4.3	5.25	3.28	3.84	1.01	0.94	0.13		4.26
Slag	37.1	45.2	1.85	6.44	0.793	5.76	0.517	1.02	0.285	0.683	0.33
Sand	97.89	0.72	0.06	0.6	0.46						0.27
BBA	43.66	24.08	3.21	7.28	10.34	2.44	1.76	2.1	1.34	1.39	2.3

**Table 2:** Mix proportions of repairing mortars (kg).

Samples	OPC	Slag	Sand	BBA	Water	NaOH
OPC/Sand	0.450	-	1.350	-	0.235	-
AAS/Sand	-	0.450	1.350	-	0.245	0.0097
AAS/BBA	-	0.450	-	1.350	0.237	0.0097

**Table 3:** Volume density and shrinkage due to drying of hardened specimens.

Mortars	Density, kg/m^3^		Shrinkage, mm/m	
	After 7 Days	After 28 Days	After 7 Days	After 28 Days
OPC/Sand	2072	2098	3.162	3.210
OPC/Sand	1859	1894	2.020	1.903
AAC/BBA	1892	1936	3.660	3.121

## Data Availability

The original contributions presented in the study are included in the article, further inquiries can be directed to the corresponding author.
